# Assessing stool quantities generated by three specific Kato-Katz thick smear templates employed in different settings

**DOI:** 10.1186/s40249-016-0150-9

**Published:** 2016-07-01

**Authors:** Andrea Leuenberger, Tatu Nassoro, Khadija Said, Lukas Fenner, George Sikalengo, Emilio Letang, Antonio Montresor, Xiao-Nong Zhou, Peter Steinmann, Hanspeter Marti, Jürg Utzinger, Stefanie Knopp

**Affiliations:** Swiss Tropical and Public Health Institute, P.O. Box, CH-4002 Basel, Switzerland; University of Basel, P.O. Box, CH-4003 Basel, Switzerland; Exercise Physiology Lab, Institute of Human Movement Sciences and Sport, ETH Zurich, Winterthurerstrase 190, CH-8057 Zurich, Switzerland; Bagamoyo Research and Training Centre, Bagamoyo District Hospital, Ifakara Health Institute, P.O. Box 74, Bagamoyo, United Republic of Tanzania; Institute of Social and Preventive Medicine, University of Bern, Finkenhubelweg 11, CH-3012 Bern, Switzerland; Ifakara Health Institute, Off Mlabani Passage, P.O. Box 53, Ifakara, United Republic of Tanzania; Department of Control of Neglected Tropical Diseases, World Health Organization, Avenue Appia 20, CH-1211 Geneva 27, Switzerland; Chinese Center for Disease Control and Prevention, National Institute of Parasitic Diseases, 207 Rui Jin Er Road, Shanghai, 200025 People’s Republic of China; Key Laboratory on Biology of Parasite and Vector, Ministry of Health, WHO Collaborating Center for Malaria, Schistosomiasis and Filariasis, 207 Rui Jin Er Road, Shanghai, 200025 People’s Republic of China; Wolfson Wellcome Biomedical Laboratories, Department of Life Sciences, Natural History Museum, Cromwell Road, London, SW7 5BD United Kingdom

**Keywords:** Accuracy, Diagnosis, Infection intensity, Kato-Katz technique, Schistosomes, Soil-transmitted helminths

## Abstract

**Background:**

The Kato-Katz technique is recommended for the diagnosis of helminth infections in epidemiological surveys, drug efficacy studies and monitoring of control interventions. We assessed the comparability of the average amount of faeces generated by three Kato-Katz templates included in test kits from two different providers.

**Methods:**

Nine hundred Kato-Katz thick smear preparations were done; 300 per kit. Empty slides, slides plus Kato-Katz template filled with stool and slides plus stool after careful removal of the template were weighed to the nearest 0.1 mg. The average amount of stool that was generated on the slide was calculated for each template, stratified by standard categories of stool consistency (i.e. mushy, soft, sausage-shaped, hard and clumpy).

**Results:**

The average amount of stool generated on slides was 40.7 mg (95 % confidence interval (*CI*): 40.0–41.4 mg), 40.3 mg (95 % *CI*: 39.7–40.9 mg) and 42.8 mg (95 % *CI*: 42.2–43.3 mg) for the standard Vestergaard Frandsen template, and two different templates from the Chinese Center for Disease Control and Prevention (China CDC), respectively. Mushy stool resulted in considerably lower average weights when the Vestergaard Frandsen (37.0 mg; 95 % *CI*: 34.9–39.0 mg) or new China CDC templates (37.4 mg; 95 % *CI*: 35.9–38.9 mg) were used, compared to the old China CDC template (42.2 mg; 95 % *CI*: 40.7–43.7 mg) and compared to other stool consistency categories.

**Conclusion:**

The average amount of stool generated by three specific Kato-Katz templates was similar (40.3–42.8 mg). Since the multiplication factor is somewhat arbitrary and small changes only have little effect on infection intensity categories, it is suggested that the standard multiplication factor of 24 should be kept for the calculation of eggs per gram of faeces for all investigated templates.

**Electronic supplementary material:**

The online version of this article (doi:10.1186/s40249-016-0150-9) contains supplementary material, which is available to authorized users.

## Multilingual abstracts

Please see Additional file 1 for translation of the abstract into six official working languages of the United Nations.

## Background

More than 1.4 billion people are infected with soil-transmitted helminths (STHs) [1] and over 250 million people are infected with *Schistosoma* spp. [2, 3]. The global burden of disease due to STH infections and schistosomiasis is estimated at 5.2 million and 3.3 million disability-adjusted life years (DALYs), respectively [4]. Marginalised communities living in tropical and subtropical areas who lack access to clean water, sanitation and hygiene are most severely affected, in particular individuals with repeated and chronic infections [3, 5, 6]. To overcome the impact of STH infections, schistosomiasis and other neglected tropical diseases, in 2012, the World Health Organization (WHO) and other partners came together in a collective effort to tackle these diseases [1, 7–9].

The Kato-Katz technique is recommended by WHO for the diagnosis and quantification of *Schistosoma mansoni*, other human-pathogenic *Schistosoma* species (e.g. *S. japonicum* and *S. mekongi*), STH and other helminth infections (e.g. food-borne trematodes) [10]. Indeed, the Kato-Katz technique is widely used in epidemiological surveys, drug efficacy studies and sentinel surveillance to determine the impact of helminth control programmes [11]. The simple performance and relatively low financial costs of the technique favour its application for field work in resource-constrained settings and in laboratories with restricted human and financial capacity [12]. By counting the number of eggs in a defined amount of stool and by multiplying the counts with a factor that allows the transformation in eggs per gram of stool (EPG), the Kato-Katz technique enables the quantification of the infection. Importantly, faecal egg counts expressed as EPG are widely used as proxy for infection intensity, which is classified by WHO into light, moderate and heavy [13, 14], and are used as indirect measure for worm burden and morbidity [8, 15]. Moreover, infection intensity categories are employed to define the frequency of preventive chemotherapy interventions in helminthiases control and elimination programmes [8, 13, 16].

Until recently, the Kato-Katz diagnostic kit that has been widely used by research groups and managers of helminthiases control programmes for drug efficacy studies, sentinel surveillance and monitoring of control programmes was manufactured by the Vestergaard Frandsen Group (Lausanne, Switzerland). In order to maintain the availability of the Kato-Katz kits for the diagnosis of helminth infections and quantifying infection intensities, WHO invited the submission of kits from other providers, including the Chinese Center for Disease Control and Prevention (China CDC). A traditional kit provided by China CDC (designated “old China CDC template”) contains a plastic template that has been used in previous research projects conducted in the People’s Republic of China and elsewhere [17–19]. Recently, China CDC has developed a new kit (designated “new China CDC template”). The plastic templates included in the Vestergaard Frandsen and the new China CDC kit have an identical cylindrical hole with a diameter of 6.0 mm and a height of 1.5 mm, and are hence supposed to deliver the same volume of stool, namely a cylinder of 42.4 mm^3^. The old China CDC kit has a height of only 1.0 mm and a hole shaped as truncated cone with a diameter of 6 mm on the top and 8 mm at the bottom side ([20], in Chinese) and is supposed to hold a volume of 38.7 mm^3^ stool. The WHO bench aids indicate that standard Kato-Katz templates with a diameter of 6.0 mm and a height of 1.5 mm generate an amount of 41.7 mg of stool [21] and studies using the old China CDC template have worked with the same approximation [17, 18]. Evidence supporting the suggested 41.7 mg and the resulting multiplication factor of 24 has, to our knowledge, not been published for any of the three Kato-Katz kits.

In the current study, we investigated the average amount of stool that remained on a microscope slide after careful removal of (i) the standard Vestergaard Frandsen template, (ii) the old China CDC template and (iii) the new China CDC template, respectively, in three different laboratories (Bagamoyo and Ifakara, United Republic of Tanzania; Basel, Switzerland), in order to provide better evidence for the application of the templates with the recommended standard multiplication factor of 24 to calculate EPG.

## Methods

### Study design and sample size

To determine the sample size for our study, we considered the multiplication factor to determine EPG as most essential. In line with changes for infection intensity categorisation for *S. mansoni*, we estimated that multiplication factors of 15 (and below) and of 35 (and above) instead of the recommended factor of 24 would result in noticeably wrong categorisations of *S. mansoni* infection intensities. Hence, we aimed to detect a significant difference between 28.6 mg and 41.7 mg and between 41.7 mg and 66.7 mg, which resulted in required sample sizes of 222 and 68, respectively. To be conservative, we decided to perform 300 weighings per template type.

The following three Kato-Katz templates were compared: (i) standard Vestergaard Frandsen template; (ii) old China CDC template; and (iii) new China CDC template. As summarised in Table 1, the three templates are small square plastic tiles (30 mm × 40 mm) with a central hole.

**Table 1:** Specification of the plastic templates included in the Kato-Katz kits provided by Vestergaard Frandsen and the Chinese Center for Disease Control and Prevention (China CDC)

Template	Vestergaard Frandsen	Old China CDC	New China CDC
Shape of hole	Cylinder	Truncated cone	Cylinder
Diameter of hole	6.0 mm	8.0 mm (bottom) 6.0 mm (top)	6.0 mm
Thickness of template	1.5 mm	1.0 mm	1.5 mm
Volume of hole	42.4 mm^3^	38.7 mm^3^	42.4 mm^3^
Manufacturer	Vestergaard Frandsen; Lausanne, Switzerland	Combined Biotech Co.; Shenzhen, P.R. China	Combined Biotech Co.; Shenzhen, P.R. China

To obtain a total of 300 weighings per template type, and in line with the pioneering study published by Katz and colleagues in 1972 [12], we selected 10 stool samples in each of three laboratories and prepared a total of 30 Kato-Katz thick smears from each stool sample, 10 with each of the three templates under investigation. Hence, in each laboratory, we performed 100 weighings per template and in total, for each template, we took 300 weighings. Stool samples were only eligible for inclusion in the study when they were of sufficient size to prepare a total of 30 Kato-Katz thick smears.

### Study settings and population

The Kato-Katz thick smear templates from Vestergaard Frandsen and China CDC were evaluated in (i) the laboratory of the Helminth Unit, Bagamoyo Research and Training Center (BRTC) of the Ifakara Health Institute (IHI) in Bagamoyo, United Republic of Tanzania (in September 2015); (ii) the laboratory of the Chronic Disease Center Ifakara (CDCI) of IHI in Ifakara, United Republic of Tanzania (in September 2015); and (iii) the Coprology Laboratory of the National Reference Centre for Imported Parasitic Diseases at the Swiss Tropical and Public Health Institute (Swiss TPH) in Basel, Switzerland (in October 2015).

Staff in the Tanzanian laboratories was well acquainted in helminth diagnostic procedures, as they regularly carry out examinations within the frame of epidemiological surveys and clinical trials [22–24]. Standard operating procedures (SOPs) and internal quality control (QC) for microscopic readings, including Kato-Katz thick smears, room and freezer temperatures and equipment for molecular analysis such as polymerase chain reaction (PCR) and enzyme-linked immunosorbent assays (ELISA) are in place. The laboratory in Basel is part of the Diagnostic Centre of Swiss TPH, which is accredited and specialised in the diagnosis of tropical diseases, including stool examinations for intestinal parasitic infections.

In the United Republic of Tanzania, stool samples were obtained from individuals within two tuberculosis (TB) cohorts, who submitted stool samples for a study assessing TB and helminth co-infections. In Switzerland, stool samples were obtained from outpatients who submitted stool samples for examination upon the doctor’s request in relation to signs and symptoms of the digestive tract. While stool samples from cohort participants in Tanzania and from outpatients in Switzerland were used to assess the weight of stool fitting into Kato-Katz templates from different manufacturers in our study, the assessment of helminth infection status and collection of health-related information from the individuals was not part of the current investigation. Diagnosis of intestinal parasitic infections was only done within the frame of the co-infection study (United Republic of Tanzania) or for expert diagnostic procedures (Switzerland).

### Ethics, consent and permissions

Stool samples used for our sub-study conducted in the two Tanzanian laboratories were obtained from individuals participating in the TB cohorts in Dar es Salaam (TB-DAR) and Ifakara addressing a wide range of research questions, including the association between TB and helminth co-infections. Ethical approval was obtained from the institutional review board of IHI (IHI/IRB/no: 04-2015) and the Medical Research Coordinating Committee of the National Institute of Medical Research (NIMR/HQ/R.8c/vol. I/357). All Tanzanian individuals who submitted stool samples were aged 18 years and above and provided written informed consent to participate in the TB cohort and co-infection study. Participants who were found infected with STHs and/or *S. mansoni* were treated according to the study protocol and in line with treatment guidelines from the United Republic of Tanzania in the frame of the TB and helminth co-infection study.

Stool samples from the sub-study carried out in Switzerland were obtained from the diagnostic laboratory of the travel clinic of Swiss TPH, where stool samples from people with a recent travel history to the global South are examined on a day-to-day basis. Here, outpatients with specific digestive signs submit stool samples for parasitological examination upon the doctor’s expert opinion. No written informed consent was requested from the outpatients. Infected individuals received a prescription for treatment from the responsible doctor of the travel clinic of Swiss TPH.

Of note, stool samples from Tanzanian and Swiss individuals were used to assess the weight of stool fitting into Kato-Katz templates from different manufacturers. However, the assessment of infection status and collection of health-related information from the individuals was not part of the current study.

### Laboratory procedures

In a first step, the consistency of each stool sample was classified according to the Bristol stool chart [25]. Diarrhoeic stool samples were not included in the study. Secondly, for the measurement of the weight of stool that remained on the microscope slide after careful removal of the kit template, each of the 300 labelled slides and of the 100 labelled plastic templates from Vestergaard Frandsen and China CDC (old and new type of the template), respectively, were weighed on a high precision scale (Ifakara: Adventurer® Analytical and Precision Scale, item no.: AR2140, series no.: 1202500496, OHaus Corporation, Parsippany, NJ, USA; Bagamoyo: ae Adam, item no.: AEP250G, series no.: AE16690611, Adam Equipment Co Ltd, Danbury, CT, USA; Basel: Mettler Toledo, item no.: AC88, series no.: 77 97 13, Mettler Instrumente AG, Greifensee, Switzerland) and recorded at the nearest 0.1 mg. Subsequently, the plastic templates were filled with stool as per standard Kato-Katz procedures [12]. For this purpose, a part of each stool sample was placed on a newspaper and covered with a nylon screen with a mesh size of 80 μm. The stool was forced through the screen using a plastic spatula and a portion of the sieved material filled in the respective template that had been placed on the weighed labelled microscope slides. With the spatula, the surface was levelled and excess stool from the edge of the template hole was carefully removed. Each slide plus the template filled with stool was weighed. Finally, the templates were carefully removed from the slides by tilting it up on the edge and lifting it vertically. The remaining stool plus slide were weighed again before the next slide was prepared. This procedure was executed by the same trained person (AL) in the three laboratories. No additional steps of the Kato-Katz method were performed.

### Data management and analysis

Stool consistency and weight measures were directly recorded in the laboratory. Data were analysed using STATA version 12 (StataCorp., College Station, TX, USA). Based on the Bristol stool scale, the stool specimens were classified into five consistency categories: mushy, soft, sausage-shaped, hard and clumpy [25, 26]. The weight of stool generated by the different templates was calculated after removal of the template to show the amount that is used for diagnosis and calculation of EPGs. The following formula was used for calculation: amount of stool form (i.e. a cylinder in case of the Vestergaard Frandsen and new China CDC templates; a truncated cone in case of the old China CDC template) = weight (labelled slide + stool)−weight (labelled slide). The arithmetic mean (AM) weight and 95 % confidence intervals (*CIs*) were calculated for the total of 300 thick smears per template and for the 100 thick smears per template in each study stetting, respectively. Moreover, to investigate the effect of stool consistency on the weight, the AM and corresponding 95 % *CIs* were calculated, stratified by stool consistency category.

## Results

### Stool sample characteristics

**Fig. 1:**
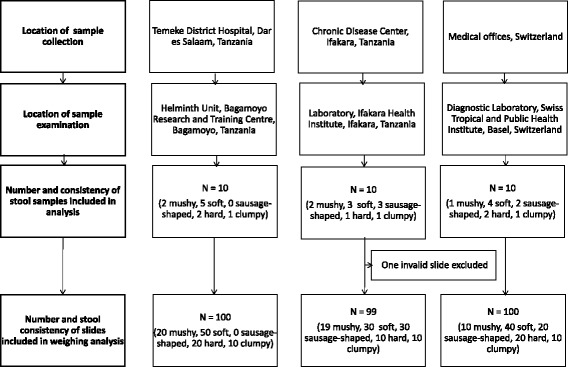
Flowchart detailing the sampling and analysis process. The location of stool sample collection and examination, and the number and stool consistency of samples and slides measured is indicated

As shown in Figure 1, in each of the three settings, 10 stool samples of sufficient size to prepare a total of 30 Kato-Katz thick smears were obtained. Among the 30 stool samples, five samples were categorised as mushy, 12 as soft, five as sausage-shaped, five as hard and the remaining three as clumpy. Overall, we measured 49 slides with stool forms prepared from mushy, 120 slides with soft, 50 slides with sausage-shaped, 50 slides with hard and 30 slides with clumpy stool. One measure with mushy stool was invalid, and hence, excluded from further analysis.

### Heterogeneity of weight of stool forms across different settings

**Fig. 2:**
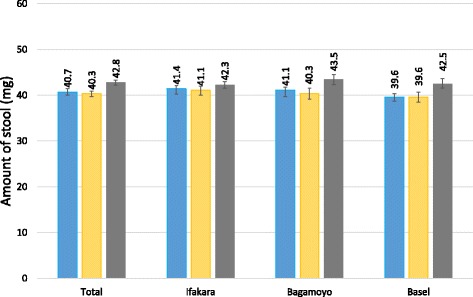
Arithmetic mean weight of stool generated on slides, stratified by study setting. The average weight of the stool forms that remained on the slides after careful removal of the Kato-Katz templates provided by Vestergaard Frandsen or the Chinese Center for Disease Control and Prevention (China CDC) is indicated, stratified by the location of the laboratory where the measuring was conducted. Blue, Vestergaard Frandsen template; yellow, new China CDC template; grey, old China CDC template; error bars represent 95 % confidence intervals

The average weight of stool produced by the three different Kato-Katz templates is indicated in Table 2. As shown in Figure 2, the average amount of stool that remained on the slides in the three settings ranged from 39.6 mg to 43.5 mg. In Ifakara, the Vestergaard Frandsen template produced an average weight of 41.4 mg, whilst the new and old China CDC templates produced average weights of 41.1 mg and 42.3 mg, respectively. In Bagamoyo, the respective average weights were 41.1 mg, 40.3 mg and 43.5 mg. Here, the average weight derived by the old China CDC template was considerably higher than the weight produced by the new China CDC template and the Vestergaard Frandsen template. In Basel, the average weight by both the Vestergaard Frandsen and the new China CDC template was 39.6 mg and the average weight obtained by the old China CDC template was 42.5 mg. The average weight by the old China CDC template was again considerably higher than the weights by the other two templates.

**Table 2:** Average weight of stool produced by Kato-Katz templates from different providers. Arithmetic mean (AM) weight and 95 % confidence intervals (*CIs*) of stool remaining on the microscope slide after removal of the Kato-Katz templates from Vestergaard Frandsen and the Chinese Center for Disease Control and Prevention (China CDC)

Template	Weight of stool remaining on the slide	
	AM mg	(95 % *CI*)
Vestergaard Frandsen	40.7	(40.0–41.4)
New China CDC	40.3	(39.7–40.9)
Old China CDC	42.8	(42.2–43.3)

### Effect of stool consistency on weight of stool form

**Fig. 3:**
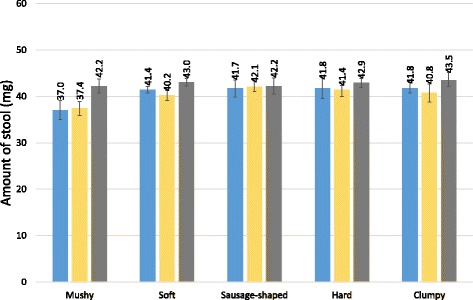
Arithmetic mean weight of stool generated on slides, stratified by stool consistency. The average weight of the stool forms that remained on the slides after removal of the Kato-Katz templates provided by Vestergaard Frandsen or the Chinese Center for Disease Control and Prevention (China CDC) is indicated, stratified by stool consistency. Blue, Vestergaard Frandsen template; yellow, new China CDC template; grey, old China CDC template; error bars represent 95 % confidence intervals

As illustrated in Figure 3, with the exception of mushy and soft stool, the consistency had only very little effect on the average weight measured for any of the three templates. The average weight of sausage-shaped, hard and clumpy stool forms ranged from 40.8 mg to 43.5 mg, regardless of the template used. Compared with the Vestergaard Frandsen and new China CDC template, the average weight of the stool form that remained on the slides with mushy and soft stool were considerably higher when the old China CDC template was employed.

## Discussion

The control of morbidity due to STH and schistosome infections and the local elimination of transmission are key targets set by WHO [7, 8, 27]. The Kato-Katz technique is recommended for quantitative diagnosis in epidemiological surveys, drug efficacy trials and sentinel surveillance of helminthiases control programmes [11, 28]. Its application has a several decade long history [29–32], but the validity of the 41.7 mg of stool that reportedly fit into the widely distributed standard plastic templates and the recommended multiplication factor of 24 has, to our knowledge, yet to be assessed. Here, we investigated the comparability of the average amount of faeces generated by three Kato-Katz templates from two providers to derive evidence for their application with the recommended standard multiplication factor of 24 to calculate EPG.

We found that the average amount of stool that remained on a microscope slide after careful removal of the template was 40.7 mg, 40.3 mg and 42.8 mg when the templates from Vestergaard Frandsen, new China CDC and old China CDC, respectively, were used. Hence, the suggested amount of 41.7 mg for the stool forms produced by these types of templates seems to be a reasonable approximation and the multiplication factor of 24 can be applied.

Our effort to reveal the origin of the suggestion of 41.7 mg generated by standard templates with a cylindrical hole with a diameter of 6.0 mm and a height of 1.5 mm shed light on the following: in early publications mentioning the Kato-Katz method, the exact amount of stool is not described precisely; instead, statements such as “a grain of rice” or a “small soybean” were found [33, 34]. The template as a volumetric device was introduced by Katz *et al.* in 1972 and thus added a means for quantification and paved the way to calculate EPGs, offering an objective measure of infection intensity. Katz and colleagues’ evaluation of the new technique included the first weighing analysis that we found in the published literature. In their seminal work, Katz and his collaborators used a thinner cardboard (1.37 mm) with a hole-diameter of 6.0 mm. While a direct calculation of the volume of the template cylinder (pi*r^2^*h, where *pi* is a constant of 3.1416, *r* is the radius and *h* is the height) results in 38.7 mm^3^, when a stool density of 1 is assumed, their examinations of 10 samples with 10 smears per sample resulted in an average weight of 43.7 mg [12]. A study conducted by Engels and colleagues showed that while their template was designed to contain 28.3 mm^3^ of stool, the average weight of examined stool was 23.0 mg [35]. In our study, the Vestergaard Frandsen and new China CDC templates were designed to produce a cylinder of 42.4 mm^3^ and the old China CDC template was expected to generate a volume of 38.7 mm^3^. Hence, as in Engels and colleagues’ investigation, in our study the average weight of the stool cylinder remaining on the slide after removal of the template was smaller than the calculated volume for the Vestergaard Frandsen and the new China CDC templates. In contrast and as in Katz *et al.*’s study, the average weight of the stool truncated cone produced by the old China CDC templates was higher than the calculated volume.

Clearly, the lower weight can be explained by a considerable amount of stool that remains in the template while it is being removed or by stool with a density below 1. While suggested as a possibility for variation in weight by Engels *et al.* [35], stool consistency showed only little effect on the weight in our study. However, mushy stool was an exception, as we observed considerably lower average measurements compared to the other stool consistencies, both for the Vestergaard Frandsen and the new China CDC templates. Mushy stool might indeed have a lower density than stool of other consistency. Moreover, the surface texture of the template might influence the amount of stool removed with the template. As pointed out elsewhere, inter- and intra-assistant differences might also contribute to the variation in weights [35]. The higher amount of stool produced with the old China CDC template might partially be a result of the conical shape of the hole, which is reported to ease the passing of stool and lifting of the slide [20].

We assume that, most likely, the suggested weight of 41.7 mg for stool forms produced by the applied templates is the result of dividing 1,000 mg by a factor of 24, which was then chosen for operational ease, but published evidence of whether this assumption is true is missing. We provide new evidence that the precise indication of 41.7 mg as average is very near the average measured with any of the three investigated templates. According to our study results, for both the Vestergaard Frandsen and new China CDC kit, one would ideally assume a weight of 40.0 mg, and hence a multiplication factor of 25 to obtain EPG estimates. On the other hand, for the old China CDC kit, a weight of 43.5 mg and a multiplication factor of 23 would result from our data. The exact multiplication factor to estimate EPGs, however, hardly influences the classification into infection intensities [35]. More important factors for reliable EPGs, and hence intensity category results, are the careful preparation of Kato-Katz thick smears and, in particular, the accurate counting of helminth eggs on a thick smear examined under a microscope [35, 36]. Since the Kato-Katz technique lacks sensitivity for the diagnosis of low-intensity infections, we recommend the examination of multiple stool samples and combining the Kato-Katz method with more sensitive diagnostic methods such as the (Mini-)FLOTAC for STHs or the point-of-care circulating cathodic antigen (POC-CCA) urine cassette test for *S. mansoni* detection in areas with low endemicity or for drug efficacy trials [22, 37–40].

## Conclusion

The average amount of stool generated by three different Kato-Katz templates was similar (40.3–42.8 mg). Since the multiplication factor is somewhat arbitrary and small changes (i.e. adaptation from 24 to 23 or 25) only have little effect on helminth infection intensity categories, it is suggested that the multiplication factor of 24 should be kept for the calculation of EPGs for all investigated templates.

## Abbreviations

AM, Arithmetic Mean; BRTC, Bagamoyo Research and Training Center; China CDC, Chinese Center for Disease Control and Prevention; CDCI, Chronic Disease Center Ifakara; DALY, disability-adjusted life year; ELISA, enzyme-linked immunosorbent assay; EPG, eggs per gram (of stool); IHI, Ifakara Health Institute; PCR, polymerase chain reaction; POC-CCA, point-of-care circulating cathodic antigen; QC, quality control; SOP, standard operating procedure; STH, soil-transmitted helminth; Swiss TPH, Swiss Tropical and Public Health Institute; TB, tuberculosis; WHO, World Health Organization;

## Additional file

Additional file 1: Multilingual abstract in the six official working languages of the United Nations. (PDF 533 kb)
