# Supplementary material for: Assessing stool quantities generated by three specific Kato-Katz thick smear templates employed in different settings
Source: Infect Dis Poverty. 2016 Jul 1;5:58. doi: 10.1186/s40249-016-0150-9 (PMC4929780; doi:10.1186/s40249-016-0150-9)
Supplement: Additional file 1: — Multilingual abstract in the six official working languages of the United Nations. (PDF 533 kb) [file 40249_2016_150_MOESM1_ESM.pdf]

Translation of the abstract into the six official working languages of the United Nations

تقييم كميات البراز الناتجة من ثلاثة قوالب كاتو-كاتز ذات مسحة سميكة محددة مستخدمة في بيئات مختلفة

أندريا وينبرجر، تاتو ناسورو، خديجة سعيد، لوكاس فينر، چورچ سيكالينجو، إميليو ليتانج، أنطونيو مونتريسور، شياو نونج تشو، بيتر شتاينمان، هانز بيتر مارتي، يورج أوتزنجر وستيفاني نوب

ملخص

خلفية: ينصح باستخدام تقنية كاتو-كاتز لتشخيص الإصابة بالديدان في المسوح الوبائية، ودراسات نجاعة الأدوية ورصد المكافحة. قمنا بتقييم إمكانية المقارنة بين متوسط كمية البراز التي تولدها ثلاثة قوالب كاتو-كاتز المدرجة في مجموعات اختبار من مصدرين مختلفين.

الطرق: أخذت 900 مسحة سميكة كاتو-كاتز. 300 في المجموعة. وتم وزن الشرائح فارغة، والشرائح بالإضافة إلى قالب كاتو-كاتز مليئة البراز والشرائح بالإضافة إلى البراز بعد إزالة متأنية من القالب إلى أقرب 0.1 مجم. تم حساب متوسط كمية البراز التي تم إنشاؤها على الشريحة لكل قالب، مرتبة حسب الفئات القياسية من قوام البراز (أي طري، لين، صلب وملتف على شكل نقانق).

النتائج: كان متوسط كمية البراز الموضوعة على شرائح المجهر 40.7 مجم (95٪ مدى الثقة (نطاق الثقة): 41.4-40.0 مجم)، 40.3 مجم (95٪ نطاق الثقة: 40.9-39.7 مجم) و42.8 مجم (95٪ نطاق الثقة: 42.2 -43.3 مجمم) لقالب فسترجارد فراندسن القياسي، وقالبان مختلفان من المركز الصيني للسيطرة على الأمراض والوقاية منها، على التوالي. ظهر البراز طري بمتوسط أوزان أقل من ذلك بكثير عند استخدام قالب فسترجارد فراندسن (37.0 مجم، 95٪ نطاق الثقة: 39.0-34.9 مجم) أو قوالب الصين CDC المركز الصيني للسيطرة على الأمراض والوقاية منها الجديدة (37.4 مجم، 95٪ نطاق الثقة: 38.9-35.9 مجم)، مقارنة مع قالب المركز الصيني للسيطرة على الأمراض والوقاية منها القديم (42.2 مجم، 95٪ نطاق الثقة: 43.7-40.7 مجم) وبالمقارنة مع فئات اتساق البراز الأخرى.

الخلاصة: إن متوسط كمية البراز التي تولدها ثلاثة قوالب كاتو-كاتز مختلفة كانت متماثلة (42.8-40.3 مجم). وحيث أن عامل الضرب اعتباطي إلى حد ما والتغيرات الصغيرة ليس لها إلا أثر ضئيل على فئات كثافة العدوى، من المقترح عامل إبقاء عامل الضرب القياسي لحساب البيض في كل جرام من البراز لجميع القوالب المفحوصة عند 24.

Translated from English version into Arabic by Mahmoud Sami, through

TRANSLATORS WITHOUT BORDERS

评估不同条件下 3 种特定的 Kato-Katz 厚涂片定量板产生的粪便量

Andrea Leuenberger, Tatu Nassoro, Khadija Said, Lukas Fenner, George Sikalengo, Emilio Letang, Antonio Montresor, Xiao-Nong Zhou, Peter Steinmann, Hanspeter Marti, Jürg Utzinger and Stefanie Knopp

摘要

引言：在涉及蠕虫感染诊断的流行病学调查、药物疗效的研究和监测控制干预措施中，改良加藤法（Kato-Katz）法是常被推荐的技术。我们比较研究了两个不同供应商提供的检测试剂盒种的 3 种 Kato-Katz 定量板的所产生的平均粪便量。

方法：共制备了 900 份 Kato-Katz 厚涂片，每种试剂盒制备 300 个。空玻片、玻片加涂粪 Kato-Katz 定量板及玻片加粪便（小心移除定量板）校准称重到接近 0.1 mg。根据粪便的标准分类方法分为不同等级（例如，糊状，软便，腊肠型，硬便和块状），计算每个定量板产生的平均粪便量。

结果：来自瑞士弗兰德森（1 种）和中国疾控中心（2 种）的定量板所产生的平均粪便量分别为 40.7 mg [95% 置信区间（*CI*）: 40.0-41.4 mg]、40.3 mg (95% *CI*: 39.7-40.9 mg)和 42.8 mg(95% *CI*: 42.2-43.3 mg)。与使用

中国疾控中心老板(42.3.4 mg; 95% *CI*: 40.7-43.7 mg)相比，使用瑞士弗兰德森(37.0 mg; 95% *CI:* 34.9-39.0 mg)和中国疾控中心新定量板(37.4 mg; 95% *CI*: 35.9-38.9 mg)时，糊状大便导致平均重量明显降低。
结论：3 种不同的 Kato-Katz 定量板产生的粪便平均值相近（40.3-42.8 mg），由于倍乘因数某些时候太过主观，而且细小改变对感染强度类型判别影响甚微，因此建议在对所有调查模板计算粪便每克卵数时应该保留标准倍乘因数 24。

Translated from English version into Chinese by Feng Xin-Yu, edited by Yang Pin, through

CHINESE CENTER FOR DISEASE CONTROL AND PREVENTION
NATIONAL INSTITUTE OF PARASITIC DISEASES

Évaluation des quantités de selles obtenues avec trois calibres de frottis en couche épaisse Kato-Katz spécifiques dans des contextes différents

Andrea Leuenberger, Tatu Nassoro, Khadija Said, Lukas Fenner, George Sikalengo, Emilio Letang, Antonio Montresor, Xiao-Nong Zhou, Peter Steinmann, Hanspeter Marti, Jürg Utzinger and Stefanie Knopp

Résumé

Contexte: La technique de Kato-Katz est recommandée pour le diagnostic des helminthiases dans les enquêtes épidémiologiques, les études sur l'efficacité des médicaments et le contrôle des interventions de lutte. Nous avons évalué la comparabilité de la quantité moyenne de selles obtenue avec trois calibres inclus dans les kits de test Kato-Katz de deux fournisseurs différents.
Méthodes: Nous avons réalisé 900 frottis en couche épaisse selon la méthode Kato-Katz, à raison de 300 par type de kit. Des lames vides, des lames avec le calibre rempli de selles et des lames garnies de selles après retrait du calibre ont été pesées à 0,1 mg près. La quantité moyenne de selles obtenue sur la lame a été calculée pour chaque calibre, en stratifiant selon les catégories standard de consistance des selles (détrempées, molles, moulées, dures et morcelées).
Résultats: La quantité moyenne de selles obtenue sur les lames de microscope était de 40,7 % (intervalle de confiance (*IC*) à 95 % : 40,0-41,4 mg), 40,3 mg (*CI* à 95 % : 39,7-40,9 mg) et 42,8 mg (*CI* à 95 % : 42,2-43,3 mg) avec le calibre standard Vestergaard Frandsen et deux calibres différents du Centre de lutte et de prévention des maladies chinois (China CDC), respectivement. Les selles liquides ont donné un poids moyen nettement inférieur avec le calibre Vestergaard Frandsen (37,0 mg ; *IC* à 95 % : 34,9-39,0 mg) ou le nouveau calibre China CDC (37,4 mg; *IC* à 95 % : 35,9-38,9 mg) par rapport à l'ancien calibre China CDC (42,2 mg ; *IC* à 95 % : 40,7-43,7 mg) et aussi par rapport aux autres catégories de consistance des selles.
Conclusion : La quantité moyenne de selles obtenue avec trois calibres différents pour la méthode Kato-Katz était similaire (entre 40,3 et 42,8 mg). Le facteur de multiplication étant souvent arbitraire et les petits changements n'ayant que peu d'effet sur les catégories d'intensité de l'infestation, nous suggérons que l'on pourrait garder le facteur standard de multiplication par 24 pour calculer la charge d'œufs par gramme de selles avec tous les calibres étudiés.

Translated from English version into French by Suzanne Assenat, through

Оценка количества кала, выполненная с применением трех определенных шаблонов для толстых мазков по Като-Кац при различных условиях

Андреа Лойенбергер, Тату Нассоро, Хадиджа Саид, Лукас Феннер, Джордж Сикаленго, Эмилио Летанг, Антонио Монтрезор, Сяо-Нонг Чжоу, Питер Штайнман, Ханспетер Марти, Юрг Утцингер и Стефани Кнопп.

Аннотация

Основные сведения: Техника Като-Кац рекомендуется для диагностики гельминтных инфекций при эпидемиологических исследованиях, изучении эффективности лекарственных средств и мониторинге мероприятий по борьбе с заболеваниями. Мы провели оценку сопоставимости среднего количества испражнений с помощью трех шаблонов по Като-Кац, входящих в состав комплектов для тестирования, предоставленных двумя различными поставщиками.

Методы: было выполнено 900 приготовлений густых мазков по Като-Кац; по 300 на комплект. Пустые стекла, стекла плюс шаблон Като-Кац с калом, а также стекла плюс кал после аккуратного удаления шаблона были взвешены с точностью до 0,1 мг. Среднее количество кала на стекле было подсчитано для каждого шаблона с разделением по стандартным категориям консистенции кала (т.е. пористый, мягкий, колбасовидный, жесткий и в виде кусков).

Результаты: Среднее количество кала на стеклах микроскопа составило 40,7 мг (доверительный интервал 95% (CI): 40,0-41,4 мг), 40,3 мг (95% CI: 39,7-40,9 мг) и 42,8 мг (95% CI: 42,2-43,3 мг) для стандартного шаблона, предоставленного компанией Vestergaard Frandsen, а также для двух различных шаблонов, предоставленных Китайским центром контроля и профилактики заболеваний (China CDC), соответственно. Пористый стул показал значительно меньший средний вес с Vestergaard Frandsen (37,0 мг; 95% CI: 34,9-39,0 мг), или были использованы новые шаблоны China CDC (37,4 мг; 95% CI: 35,9-38,9 мг) по сравнению со старым шаблоном China CDC (42,2 мг; 95% CI: 40,7-43,7 мг) и по сравнению с другими категориями консистенции кала.

Заключение: Среднее количество кала по трем различным шаблонам Като-Кац было подобным (40,3-42,8 мг). Поскольку коэффициент умножения несколько произволен и небольшие изменения влияют на категории интенсивности инфекции лишь незначительно, предполагается, что стандартный коэффициент умножения 24 должен быть сохранен для подсчета яиц на грамм фекалий для всех исследованных шаблонов.

Translated from English version into Russian by Larysa K., through

Evaluación de cantidades de deposiciones generadas mediante tres métodos de frotis grueso Kato-Katz empleados en diferentes configuraciones

Andrea Leuenberger, Tatu Nassoro, Khadija Said, Lukas Fenner, George Sikalengo, Emilio Letang, Antonio Montresor, Xiao-Nong Zhou, Peter Steinmann, Hanspeter Marti, Jürg Utzinger y Stefanie Knopp

Resumen

Información de referencia: La técnica Kato-Katz se recomienda para el diagnóstico de infecciones de helmintos en estudios epidemiológicos, estudios de eficacia de medicamentos y supervisión de intervenciones de control. Hemos evaluado una comparativa entre la cantidad media de heces generadas por tres modelos Kato-Katz incluidos en kits de test de cuatro proveedores diferentes.

Métodos: Se realizaron 900 preparaciones de frotis grueso Kato-Katz; 300 por kit. Se pesaron portaobjetos vacíos, portaobjetos con método Kato-Katz con deposiciones y portaobjetos con deposiciones tras retirar con cuidado el cubreobjetos, con una precisión de 0,1 mg. Se calculó la cantidad media de deposiciones que se generó sobre el portaobjetos para cada método, dividido por categorías estándar de consistencia de las deposiciones (p.ej., muy blanda, blanda, en forma de salchicha, dura y grumosa).

Resultados: La cantidad media de deposiciones generadas en los portaobjetos del microscopio fue de 40,7 mg (intervalo de confianza del 95% (IC): 40,0-41,4 mg), 40,3 mg (CI del 95%: 39,7-40,9 mg) y 42,8 mg (CI del 95%: 42,2-43,3 mg) para el método Vestergaard Frandsen estándar y dos métodos diferentes del Centro Chino de Control y Prevención de Enfermedades, respectivamente. Las deposiciones muy blandas dieron como resultado unos pesos medios considerablemente inferiores cuando se utilizaron los métodos de Vestergaard Frandsen (37,0 mg; CI del 95%: 34,9-39,0 mg) o los nuevos métodos chinos (37,4 mg; CI del 95%: 35,9-38,9 mg) al compararlos con el viejo método chino (42,2 mg; CI del 95%: 40,7-43,7 mg) y con otras categorías de consistencia de los deposiciones.

Conclusión: La cantidad media de deposiciones generadas mediante tres métodos Kato-Katz diferentes fue similar (40,3-42,8 mg). Dado que el factor de multiplicación es un tanto arbitrario y las pequeñas variaciones tienen poco efecto sobre las categorías de intensidad de la infección, se sugiere mantener un factor de multiplicación estándar de 24 para el cálculo de los huevos por gramo de heces para todos los métodos investigados.

Translated from English version into Spanish by SergioLorenzi, through

TRANSLATORS
WITHOUT BORDERS
